# A Propionate Metabolism‐Based Gene Signature Reveals Immunogenomic and Transcriptomic Determinants of Prognosis in Glioblastoma Through Multiomics Integration

**DOI:** 10.1155/humu/6998123

**Published:** 2026-05-16

**Authors:** Mengtong Li, Jiayi Liu, Zichen Liu, Xia Liu, Peng Lun

**Affiliations:** ^1^ Department of Neurosurgery, The Affiliated Hospital of Qingdao University, Qingdao, Shandong, China, qdu.edu.cn

**Keywords:** glioblastoma, multiomics integration, prognostic signature, propionate metabolism, tumor microenvironment

## Abstract

Glioblastoma (GBM) remains a lethal brain tumor with limited prognostic tools. Metabolic reprogramming, particularly in understudied pathways like propionate metabolism, may offer new biomarkers. Here, we identified a novel prognostic signature based on seven propionate metabolism‐related genes (SLC9A1, ELANE, ACADS, SOAT2, MYD88, ADSL, and BMP2) from the TCGA‐GBM cohort. A risk scoring model was constructed via LASSO Cox regression effectively stratified patients into high‐ and low‐risk groups with significant survival differences, which was also validated in independent GEO datasets. Multiomics analysis revealed that the high‐risk group was associated with an immunosuppressive microenvironment, characterized by increased immune checkpoint expression and distinct immune cell infiltration. Mutational profiling showed a strong association with key driver alterations, including enrichment of RB1 mutations in high‐risk and IDH1 mutations in low‐risk patients. Single‐cell RNA‐seq (scRNA‐seq) analysis confirmed the specific enrichment of signature genes within malignant cells, and coexpression network analysis (hdWGCNA) further linked the high‐risk phenotype to transcriptional modules. In conclusion, we established and validated a robust metabolic gene signature that not only predicts prognosis but also delineates a high‐risk GBM subtype defined by integrated metabolic, immunogenomic, and transcriptional features, providing new insights into the determinants of GBM aggressiveness.

## 1. Introduction

Glioblastoma multiforme (GBM) is the most common and aggressive primary malignant brain tumor in adults, characterized by relentless proliferation, diffuse infiltration, and profound resistance to conventional therapies, with a dismal median overall survival of approximately 15 months [1, 2]. Despite extensive genomic and transcriptomic characterization efforts, such as those by The Cancer Genome Atlas (TCGA) [3], which have identified key molecular subtypes and recurrent driver alterations. However, the clinical translation of these findings into effective, personalized treatment strategies remains limited [1, 4]. This gap underscores the urgent need for robust, biologically informed prognostic biomarkers that can more accurately stratify patients and reveal novel therapeutic vulnerabilities.

A hallmark of cancer, including GBM, is metabolic reprogramming, which fuels rapid proliferation and reshapes tumor microenvironment [5, 6]. Although central carbon metabolism pathways like glycolysis and glutaminolysis have been extensively studied [6, 7], the role of specific micronutrient metabolic pathways, such as propionate metabolism, in GBM pathogenesis and prognosis is less defined. Propionate metabolism, involved in short‐chain fatty acid (SCFA) processing and methylmalonyl‐CoA pathways, intersects with crucial cellular processes including lipogenesis and the tricarboxylic acid (TCA) cycle [8]. Dysregulation in this metabolic axis could therefore significantly impact tumor bioenergetics and progression, presenting a promising yet underexplored avenue for biomarker discovery [7]. Propionate metabolism was specifically selected for three reasons. First, although central carbon metabolism (e.g., glycolysis and glutaminolysis) has been extensively studied in GBM, the role of SCFA pathways such as propionate metabolism remains largely unexplored. Second, SCFAs can influence histone acetylation and immune cell function, suggesting a potential link to the GBM tumor microenvironment. Third, our preliminary screening of metabolic pathways in TCGA‐GBM data revealed that propionate metabolism‐related genes (PMRGs) were significantly associated with patient survival, motivating a focused investigation.

Here, we hypothesized that genes involved in propionate metabolism hold prognostic value and are embedded in broader transcriptional networks influencing GBM malignancy. To test this, we conducted a comprehensive, multiomics investigation. First, we systematically identified PMRGs with prognostic significance from the TCGA‐GBM cohort. Using LASSO Cox regression, we constructed and validated a novel risk signature based on seven core PMRGs. We further explored the biological underpinnings of this signature through functional enrichment, immune cell infiltration analysis using CIBERSORT algorithm, and somatic mutational profiling comparing low‐ and high‐risk groups. To dissect cellular heterogeneity and coexpression networks at single‐cell resolution, we performed single‐cell RNA sequencing (scRNA‐seq) analysis coupled with hdWGCNA on primary GBM samples, focusing on malignant cell states. Finally, we investigated the upstream regulatory landscape, including transcription factors and microRNAs (miRNAs), targeting the signature genes. This integrative study is aimed at establishing a reliable metabolic gene‐based prognostic model for GBM and elucidate its associated immunogenomic and transcriptional features, providing new insights into the metabolic determinants of GBM aggressiveness.

## 2. Materials and Methods

### 2.1. Data Acquisition

TCGA database (https://portal.gdc.cancer.gov/) is currently the largest database of cancer gene information, including gene expression data, miRNA expression data, copy number variation, DNA methylation, SNP, and other data. We downloaded the original mRNA expression data of processed GBM, including normal group (*n* = 5), tumor group (*n* = 170). The Series Matrix File data file of GSE43378 was downloaded from the NCBI GEO public database, the annotation platform is GPL570, a total of 50 patients with complete expression profiles and survival information were extracted [9]; the Series Matrix File data file of GSE74187 (the annotation platform is GPL6480) containing 60 patient data with complete expression profiles and survival information was also downloaded [10]. The scRNA‐seq data of nine glioma patients were obtained from the GSE138794 dataset [11]. In addition, the WES data of the MSK GBM cohort were obtained from the cBioPortal database [12].

### 2.2. Analysis of GO and KEGG Functions

Functional annotation of prognostic genes was performed using the R package “ClusterProfiler” to comprehensively explore the functional correlation of these prognostic genes [13]. Gene Ontology (GO) and Kyoto Encyclopedia of Genes and Genomes (KEGG) were used to assess relevant functional categories. GO‐ and KEGG‐enriched pathways with both *p* value and *q*‐value less than 0.05 were considered as significant categories.

### 2.3. Model Construction and Prognosis

The genes related to propionate metabolism were selected, and the prognostic model was further constructed by lasso regression. After incorporating the expression values of each specific gene, a risk score formula was established for each patient and weighted with its estimated regression coefficient in the lasso regression analysis. LASSO Cox regression was performed using the glmnet R package with 10‐fold cross‐validation. The optimal penalty parameter *λ* was chosen as *λ*.min (the value that gave the minimum mean cross‐validated partial likelihood deviance). According to the risk score formula, patients were divided into low‐risk group and high‐risk group with the median risk score value as the cutoff point. Survival differences between the two groups were assessed by Kaplan–Meier and compared using the log‐rank statistical method. The proportional hazards assumption for the Cox regression models was tested using the Schoenfeld individual test, and a global test was also performed. Lasso regression analysis and stratified analysis were used to test the role of risk score in predicting patient prognosis. The ROC curve was used to study the accuracy of model prediction.

### 2.4. Immune Cell Infiltration Analysis

The CIBERSORT method is a widely used method for the evaluation of immune cell types in the microenvironment. The method is based on the principle of support vector regression, and deconvolution analysis is performed on the expression matrix of immune cell subtypes. It contains 547 biomarkers that distinguish 22 human immune cell phenotypes, including T cells, B cells, plasma cells, and myeloid cell subsets. In this study, the CIBERSORT algorithm was used to analyze the patient data to infer the relative proportion of 22 types of immune infiltrating cells, and to perform spearman correlation analysis on gene expression and immune cell content [14]. All *p* values for immune cell comparisons were adjusted for multiple testing using the Benjamini–Hochberg method; an adjusted *p* value (FDR) < 0.05 was considered significant.

### 2.5. GSEA Analysis

According to the high and low risk of the model, the patients were divided into high‐ and low‐risk groups, and the differences in signaling pathways between the two groups were further analyzed by GSEA [15]. The background gene set is an annotated gene set downloaded from the MSigDB database [16] as the annotated gene set of the subtype pathway, and the differential expression analysis of the pathways between subtypes is performed, and the gene set that is significantly enriched according to the consistency score (adjusted *p* value is less than 0.05). GSEA analysis is often used to explore the close combination of tumor classification and biological significance.

### 2.6. Nomogram Model Construction

Nomogram is based on regression analysis, according to the expression level of genes and clinical symptoms, and then uses the line segment with scale to draw on the same plane according to a certain ratio, to express the interaction between variables in the prediction model relation. By constructing a regression model, according to the degree of contribution of each influencing factor in the model to the outcome variable (the size of the regression coefficient), assign a score to each value level of each influencing factor, and then add up the scores to obtain the total score. Thus, the predicted value is calculated.

### 2.7. Regulatory Network Analysis of Model Genes

In this study, the R package “RcisTarget” was used to predict transcription factors. All calculations performed by RcisTarget are based on motifs. The normalized enrichment score (NES) of a motif depends on the total number of motifs in the database. In addition to motifs annotated by source data, we infer further annotation files based on motif similarity and gene sequence. The first step in estimating the overexpression of each motif across a gene set is to calculate the area under the curve (AUC) for each motif–motif‐set pair. This was performed based on the recovery curve calculation of the gene set versus motif ordering. The NES of each motif is calculated from the AUC distribution of all motifs in the gene set. We use rcitarget.hg19.motifdb.cisbpont.500 bp for the gene‐motif rankings database.

### 2.8. miRNA‐mRNA Network Construction

miRNAs are small noncoding RNAs that have been shown to regulate gene expression by promoting the degradation of mRNAs or inhibiting the translation of mRNAs. Therefore, we further analyzed whether some miRNAs in key genes regulate the transcription or degradation of some dangerous genes. We obtained miRNAs related to key genes through the mircode database, and visualized the miRNA network of genes through Cytoscape software.

### 2.9. scRNA‐Seq Analysis

First, the data was processed through the Seurat R package [17, 18], and the positional relationship between each cluster was obtained by using the tSNE algorithm analysis; the clusters were annotated through the celldex package, and some cells that were important for tumorigenesis were annotated. Finally, we extract the marker genes of each cell subtype from the single‐cell expression profile by setting the logfc.threshold parameter of FindAllMarkers to 1. Coexpression network analysis was performed on the single‐cell RNA‐seq data using the hdWGCNA R package [19]. Briefly, cells belonging to the major lineages of interest were subset from the integrated Seurat object. To mitigate sparsity, scRNA‐seq counts were aggregated by different groups to create a pseudobulk expression matrix. A soft‐power threshold was chosen to approximate scale‐free topology. Subsequently, a signed topological overlap matrix (TOM) was constructed, and genes were hierarchically clustered into coexpression modules using dynamic tree cutting. Module eigengenes were calculated and correlated with key sample metadata to identify biologically relevant modules. Functional enrichment analysis for GO terms and pathways within significant modules was conducted using the clusterProfiler R package.

### 2.10. Mutational Analysis

Somatic mutation analysis was performed using the TCGA‐GBM dataset. Publicly available Mutation Annotation Format (MAF) files were obtained from the Genomic Data Commons (GDC) portal. All subsequent analyses and visualizations were conducted with the maftools R package [20]. The comprehensive mutational landscape was summarized and visualized using the oncoplot function, depicting the frequently mutated genes. To interrogate the pattern and localization of variants within key driver genes, lollipopPlot was employed, mapping mutations onto the corresponding protein domains. Furthermore, to identify potential cooperative or mutually exclusive somatic interactions between significantly mutated genes, pairwise Fisher′s exact tests were performed using the somaticInteractions function. Interactions were considered statistically significant after adjusting for multiple testing using the Benjamini–Hochberg method (FDR < 0.05).

### 2.11. Statistical Analysis

All statistical analyzes were performed using R software, and *p* value < 0.05 or *q*‐value < 0.05 was considered statistically significant.

## 3. Results

### 3.1. Identification of Prognostic Genes Related to Propionate Metabolism in the GBM Cohort

**Figure 1:**
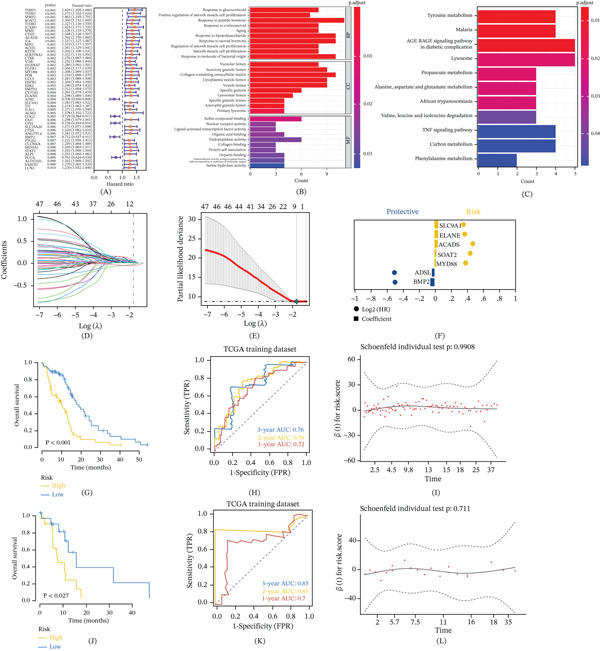
Identification and prognostic validation of a propionate metabolism‐related gene signature in GBM. (A) Univariate Cox regression analysis identified 47 prognosis‐related genes from propionate metabolism gene sets. (B–C) Functional enrichment analysis (GO and KEGG) of the 47 prognosis‐related genes. (D–E) The LASSO Cox regression algorithm was applied to screen seven hub genes (SLC9A1, ELANE, ACADS, SOAT2, MYD88, ADSL, and BMP2) from the prognostic gene set. (F) Coefficients of the seven signature genes in the LASSO model. (G) Kaplan–Meier survival analysis compares the overall survival between high‐risk and low‐risk groups in the TCGA training cohort. (H–I) Time‐dependent ROC curves assess the predictive accuracy of the risk model at 1, 2, and 3 years in the training set. (J–L) Validation of the model′s prognostic performance using Kaplan–Meier curves and ROC analysis in the TCGA testing cohort.

We downloaded the processed original mRNA expression data of GBM in the TCGA database, obtained a total of 2849 gene sets of Propionate metabolism genes through the GeneCards database (https://www.genecards.org/). The relevance score in GeneCards integrates multiple lines of evidence (e.g., protein interactions, text mining) to indicate the strength of gene–pathway association; a threshold of > 5 was applied to select moderately‐to‐highly confident PMRGs. A total of 924 candidate genes in GBM were screened using Cox univariate regression. The results showed that a total of 47 prognosis‐related genes (*p* value < 0.01) were screened out by Cox univariate regression (Figure 1A). We further conducted pathway enrichment analysis on 47 prognosis‐related genes. GO results showed that the genes were mainly enriched in response to glucocorticoid, positive regulation of smooth muscle cell proliferation, and response to peptide hormone pathways (Figure 1B). KEGG results showed that genes were mainly enriched in pathways such as tyrosine metabolism, lysosome, and propanoate metabolism (Figure 1C). To further identify the key genes in the prognostic gene set, we collected clinical information of glioma patients and screened seven feature genes, namely SLC9A1, ELANE, ACADS, SOAT2 (sterol O‐acyltransferase 2), MYD88, ADSL (adenylosuccinate lyase), and BMP2, by the Lasso regression feature selection algorithm (Figure 1D,E). Among them, SLC9A1, ELANE, ACADS, SOAT2 and MYD88 were risk factors (hazard ratio > 1), ADSL and BMP2 were protective factors (hazard ratio < 1). All expression values were log2‐transformed TPM (transcripts per million) from the TCGA‐GBM dataset. The coefficient of biomarkers was shown in Figure 1F. We randomly divided the patients into a training set and a validation set at a ratio of 4:1, and obtained the best risk score value corresponding to each sample through lasso regression analysis with the calculation formula: Risk Score = BMP2 × (−0.050423172) + ADSL × (−0.032210576) + MYD88 × 0.018755009 + SOAT2 × 0.023749711 + ACADS × 0.041906453 + ELANE × 0.046299917 + SLC9A1 × 0.048508023. Patients were divided into high‐risk and low‐risk groups according to risk scores and analyzed using Kaplan–Meier curve training. Survival rates were significantly different between the two risk groups (Figure 1G). It was determined that the survival risk model could be used as a prognostic model if the area under the ROC curve (AUC value) of 1–3 years was greater than 0.6 (Figure 1H,I). In addition, the results of the KM curves and ROC curves of the test dataset are consistent with those of the training dataset (Figure 1J–L).

### 3.2. Validation of the Established Risk Model in Independent Datasets

**Figure 2:**
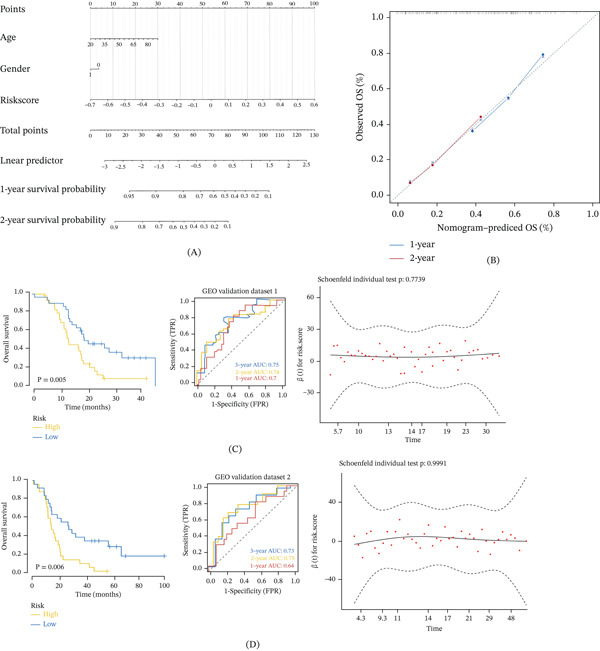
Independent validation and clinical applicability of the risk model. (A) A nomogram incorporates the risk score for predicting 1‐ and 2‐year overall survival of GBM patients. (B) Calibration curves of the nomogram for 1‐year and 2‐year survival prediction. (C) Kaplan–Meier survival analysis validates the prognostic stratification of the model in an independent GEO cohort (GSE43378). (D) ROC curve analysis demonstrates the predictive accuracy of the model in another independent GEO cohort (GSE74187).

The samples are divided into high‐risk and low‐risk groups by the median value of the riskscore value, and the results of their regression analysis are displayed in the form of a nomogram. The results of the logistic regression analysis show that in all our samples, the riskscore value has a significant contribution to the scoring process of the nomogram prediction model (Figure 2A). At the same time, we also performed calibration analysis on the 1‐ and 2‐year periods of GBM, and we found that the prediction results were favorable (Figure 2B). Then we downloaded the processed data of GBM patients with survival data in the GEO database (GSE43378 and GSE74187), predicted the clinical classification of GBM patients in the GEO database according to the model, and evaluated the survival difference between the two groups through Kaplan–Meier analysis, and explore the prediction model stability. The results showed that the overall survival of the high‐risk group in the GSE43378 dataset was significantly lower than that of the low‐risk group (*p* = 0.005, Figure 2C). To verify the accuracy of the model, we used another external dataset (GSE74187) to analyze the ROC curve of the model, and the results showed that the risk model had a significant predictive effect on the prognosis of patients (Figure 2D).

### 3.3. Expression Landscape and Transcriptional Regulation Analysis of the Seven Hub Genes

**Figure 3:**
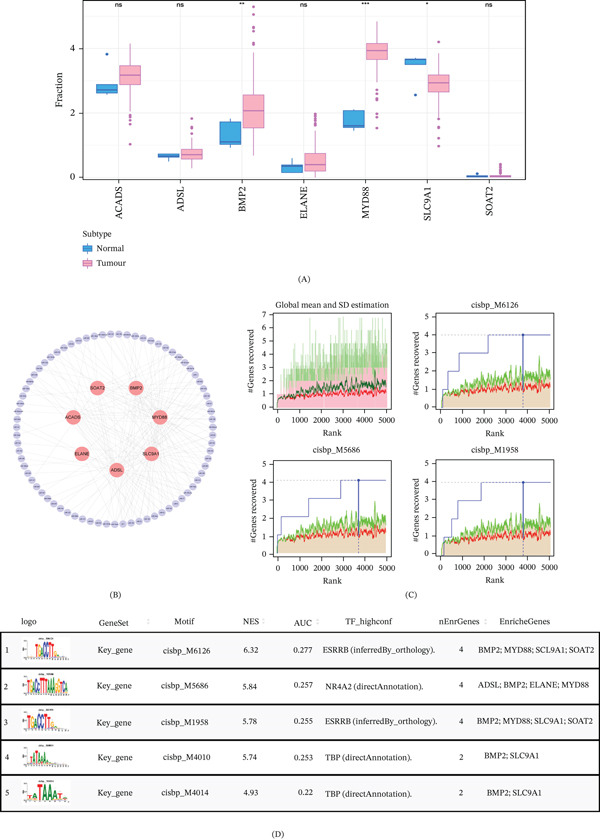
Expression patterns and transcriptional regulation of the seven hub genes. (A) The mRNA expression levels of the seven signature genes in GBM tumor tissues compared with normal brain tissues from the TCGA cohort. (B) A miRNA‐mRNA regulatory network visualizes potential interactions between 75 miRNAs and the seven hub genes, constructed using Cytoscape. (C) Cumulative recovery curves from RcisTarget analysis identifies enriched transcription factor motifs among the hub genes. (D) Detailed visualization of all significantly enriched motifs and their corresponding potential transcription factors for the model genes. ns: not significant; ∗*p* < 0.05; ∗∗*p* < 0.01; *p* < 0.001.

We analyzed the expression of signature genes in TCGA‐GBM samples. Figure 3A shows that the mRNA expression levels of BMP2 and MYD88 were significantly upregulated in the tumor samples, whereas the mRNA expression level of SLC9A1 was significantly decreased. Furthermore, we explored miRNAs that potentially target seven hub genes using the miRcode database, and we obtained 75 miRNAs, and visualized 174 mRNA–miRNA relationship pairs using Cytoscape (Figure 3B). We found that all seven hub genes are controlled by the same transcription factors and other coregulatory processes. Therefore, enrichment analysis was performed for these transcription factors using cumulative recovery curves (Figure 3C). The results showed that the motif annotation was cisbp_M6126. Among them, four key genes were enriched in this motif, namely BMP2, MYD88, SLC9A1, and SOAT2. In detail, we display all enriched motifs for model genes and the corresponding transcription factors (Figure 3D).

### 3.4. Distinct Patterns of Immune Features Were Observed Between Different Risk Groups in GBM

**Figure 4:**
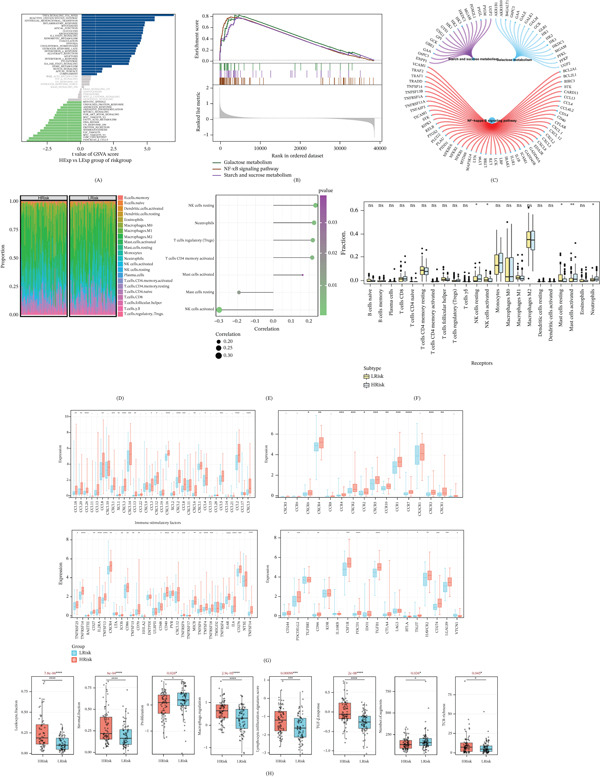
Distinct immune microenvironment features between high‐risk and low‐risk GBM groups. (A) GSVA analysis revealing differentially enriched hallmark pathways between the two risk groups. (B–C) Representative GSEA plots showing significantly enriched KEGG pathways in the high‐risk group. (D) Stacked bar plot displays the relative proportions of 22 immune cell types inferred by CIBERSORT in each sample. (E–F) Violin plots compare the infiltration levels of significantly different immune cell subsets between risk groups. (G) Heatmap shows the expression patterns of differentially expressed immune‐related genes (chemokines, immunostimulators, immunosuppressors, and receptors) across risk groups. (H) Comparison of tumor microenvironment scores (ESTIMATE, immune, and stromal) and other immunogenic features between high‐ and low‐risk patients. ns: not significant; ∗*p* < 0.05; ∗∗*p* < 0.01; *p* < 0.001; ∗∗∗∗*p* < 0.0001.

We investigated the specific signaling pathways involved in the high‐low risk correlation model to explore the underlying molecular mechanisms by which risk score affects tumor progression. GSVA results showed that the differential pathways between the two groups were mainly enriched in TNFA signaling via NFKB, reactive oxygen species (ROS) pathway, epithelial mesenchymal transition and other signaling pathways (Figure 4A). The results of GSEA showed that the involved pathways were galactose metabolism, NF − kappa B signaling pathway, Starch and sucrose metabolism (Figure 4B,C). The immune infiltration landscape of 22 immune cell types in the low‐risk and high‐risk samples was shown in the stacked plot (Figure 4D). There was a significant increase in the expressions of activated mast cells, resting NK cells and neutrophils in high‐risk groups. However, there was a significant decrease in the expression of resting mast cells and activated NK cells in high‐risk groups (Figure 4E,F). Furthermore, we found five significantly different immune cells, 19 significantly different immune‐related chemokines, 12 significantly different immunosuppressants, 23 significantly different immunostimulatory factors and 11 significantly different immune receptors across risk groups. In addition, most immune‐regulated genes (immune‐related chemokines, immune‐inhibitory factors, immune‐stimulatory factors, immunoreceptors, etc.) were highly expressed in the high‐risk group (Figure 4G). In addition, we found that leukocyte fraction, stromal fraction, macrophage regulation, lymphocyte infiltration, TGF‐*β* response, and TCR richness are significantly elevated in the high‐risk group, whereas the proliferation and numbers of segments are significantly elevated in the low‐risk group (Figure 4H). Based on these findings, patients in the high‐risk group experience more complex immunomodulatory processes, higher levels of immune evasion, and therefore may respond less effectively to immunotherapy.

### 3.5. Distinct Mutation Patterns Were Observed Between Different Risk Groups

**Figure 5:**
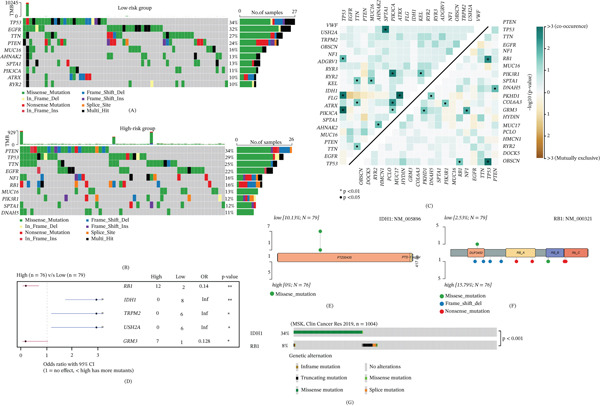
Comparative somatic mutational landscape across different risk groups. (A–B) Oncoplots summarizes the top frequently mutated genes and their variant classifications in the (A) low‐risk and (B) high‐risk cohorts. (C) Pairwise somatic interaction analysis depicts significant co‐occurrence and mutual exclusivity patterns among driver genes within each risk group. (D) Forest plot shows differentially mutated genes between risk groups, with odds ratios and statistical significance. Symbol asterisks “∗” means *p* < 0.05; “∗∗” means *p* < 0.01. (E–F) Lollipop plots illustrate the distribution and types of somatic mutations within key driver genes *IDH1* and *RB1*. (G) Validation of the mutually exclusive pattern between *IDH1* and *RB1* mutations in an independent MSK cohort (Fisher′s exact test *p* < 0.001; *n* = 1004).

To delineate the somatic mutational landscapes associated with risk stratification in GBM, we performed comprehensive mutational profiling comparing the low‐risk and high‐risk cohorts, revealing distinct genomic architectures between groups (Figure 5A,B). Pairwise co‐occurrence analysis revealed significant mutual exclusivity and comutation patterns among driver genes, with distinct oncogenic interaction networks operative across risk groups (Figure 5C). Forest plot analysis of differentially mutated genes identified significant enrichment of *RB1* alterations in the high‐risk group (15.79% vs. 2.53% in low‐risk; odds ratio [OR] = 0.14, 95*%* confidence interval excluding 1, *p* < 0.01), whereas *IDH1* mutations were exclusively detected in the low‐risk group (10.13% vs. 0%, *p* < 0.01), alongside *TRPM2* and *USH2A* which also demonstrated low‐risk predilection (Figure 5D). Protein domain mapping through lollipop plots illustrated that *IDH1* mutations clustered at the canonical R132 hotspot within the isocitrate dehydrogenase homodimerization domain, characteristic of neomorphic enzymatic activity, whereas *RB1* mutations were dispersed across functional domains including DUF3452, RB_A, and RB_B regions, encompassing both missense and truncating variants suggestive of loss‐of‐function mechanisms contributing to cell cycle dysregulation (Figure 5E,F). Importantly, these mutational signatures were corroborated in an independent validation cohort (MSK, *n* = 1004), confirming the presence of *IDH1* (34%) and *RB1* (8%) alterations at comparable frequencies, and shows the mutually exclusive feature of the two driver genes (Figure 5G). Collectively, these findings establish that genomic risk stratification delineates biologically distinct GBM subtypes characterized by divergent mutational profiles, specifically implicating *IDH1*‐driven metabolic dysregulation in low‐risk tumors and *RB1*‐mediated tumor suppressor loss in high‐risk progression, thereby offering potential prognostic biomarkers and targeted therapeutic strategies.

### 3.6. Single‐Cell Heterogeneity and Coexpression Networks in Malignant Cells of GBM

**Figure 6:**
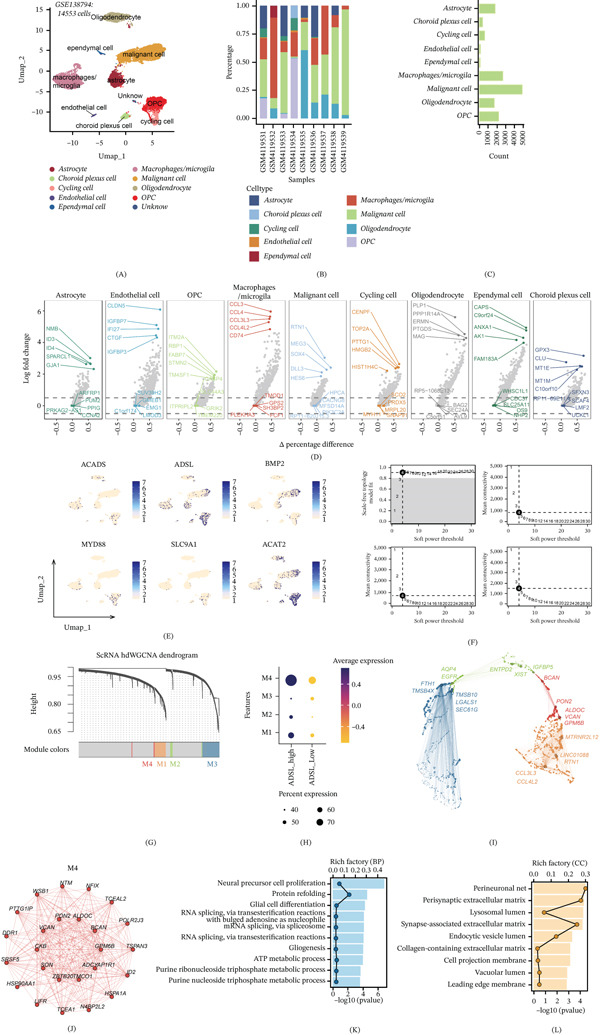
Single‐cell transcriptomic analysis and coexpression networks in GBM. (A) UMAP visualization of 14,553 single cells from GBM samples, colored by major cell clusters. (B–C) UMAP plots show the distribution of cells by (B) sample origin and (C) annotated cell types, including malignant cells, astrocytes, OPCs, and microglia/macrophages. (D) Dot plots show the expression of canonical marker genes for each identified cell type. (E) Feature plots visualize the expression distribution of the six signature genes (*ACADS, ADSL, BMP2, MYD88, SLC9A1,* and *SOAT2*) across the UMAP embedding. (F) Analysis of scale‐free topology and mean connectivity for selecting the optimal soft‐power threshold (*β* = 4) in hdWGCNA. (G) Hierarchical clustering dendrogram of genes based on topological overlap, with assigned module colors indicated. (H) Module eigengene clustering dendrogram shows the relationships among the four identified coexpression modules (M1–M4). (I) Network visualization of gene connections within a representative module. (J) Correlation heat map between module eigengenes and the trait of interest (ADSL‐high vs. ADSL‐low malignant cells). (K–L) Functional enrichment analysis (Gene Ontology) of the ADSL‐associated module M4 for (K) biological processes and (L) cellular components.

To dissect the cellular heterogeneity and transcriptional programs associated with ADSL expression in glioblastoma, we performed scRNA‐seq analysis on 14,553 cells from GBM specimens (Figure 6A). UMAP clustering identified diverse cell populations, including malignant cells, astrocytes, oligodendrocyte precursor cells (OPC), and microglia/macrophages (Figure 6A–C), with distinct lineage‐specific marker genes delineating each cluster (Figure 6D). ADSL was selected as the stratification marker because it exhibited the most prominent differential expression between malignant and nonmalignant cells among the seven signature genes (feature plot not shown), and as a protective factor in the risk model, its high expression may define a transcriptionally distinct malignant cell state with potential prognostic relevance. Notably, feature plots revealed enriched ADSL expression specifically within the malignant cell compartment, alongside coexpression patterns with ACADS, ADSL, BMP2, MYD88, SLC9A1, and ACAT2 (Figure 6E), suggesting a metabolic reprogramming signature in these tumor cells. To investigate the transcriptional networks driven by ADSL, we performed hdWGCNA on malignant cells stratified by ADSL expression (ADSL‐high vs. ADSL‐low). Topology analysis identified a soft power threshold of *β* = 4 as optimal for network construction, ensuring scale‐free topology and adequate connectivity (Figure 6F). Hierarchical clustering delineated four distinct gene coexpression modules (M1–M4) based on eigengene relatedness (Figure 6G,H). Module visualization through network plots illustrated the interconnectivity of genes within each module (Figure 6I), with module M4 demonstrating the strongest correlation with the ADSL‐high malignant cell phenotype (Figure 6J). GO analysis of the M4 module revealed significant enrichment in biological processes associated with neural precursor cell proliferation, gliogenesis, and RNA splicing (Figure 6K), whereas cellular component analysis highlighted perineuronal nets, extracellular matrix constituents, and endocytic vesicle lumens (Figure 6L), implicating ADSL‐driven programs in regulating the tumor microenvironment and malignant progression through metabolic‐transcriptional coupling.

## 4. Discussion

In this study, we have developed and comprehensively validated a novel prognostic signature for GBM based on the expression of seven PMRGs: *SLC9A1, ELANE, ACADS, SOAT2, MYD88, ADSL*, and *BMP2*. This signature effectively stratified GBM patients into distinct risk groups with significant survival differences in both the TCGA discovery cohort and independent GEO validation cohorts. Beyond its prognostic utility, our multiomics integrative analysis revealed that this metabolic gene signature is intertwined with specific immunological landscapes, somatic mutation profiles, and transcriptomic networks at single‐cell resolution, offering a holistic view of its biological underpinnings in GBM pathogenesis.

The core finding of our work is the identification of a robust metabolic determinant of patient outcome. Although metabolic reprogramming is a recognized cancer hallmark, most studies in GBM have focused on glycolysis, oxidative phosphorylation, or glutaminolysis. Our focus on propionate metabolism, a pathway involved in SCFA catabolism and propionyl‐CoA metabolism, uncovers a previously underappreciated metabolic layer. The signature genes themselves encode proteins with diverse functions, from fatty acid beta‐oxidation (*ACADS*) and cholesterol esterification (*SOAT2*) to innate immune signaling (*MYD88*) and bone morphogenesis (*BMP2*). This suggests that the “propionate metabolism” signature may not represent a linear pathway but rather a coordinated metabolic–immune‐transcriptional node. The strong association of the high‐risk score with pathways like epithelial‐mesenchymal transition (EMT), TNF‐*α*/NF‐*κ*B signaling, and ROS pathways further positions this signature within established axes of therapy resistance and tumor aggression.

Among the seven signature genes, several have known biological roles. *SLC9A1* (sodium‐hydrogen exchanger 1) regulates pH homeostasis and has been linked to invasion and chemoresistance in GBM [21]; pharmacological inhibitors such as amiloride derivatives exist but are not clinically approved for GBM. *MYD88* is a central adaptor in TLR/IL‐1R signaling; its activation promotes NF‐*κ*B‐mediated inflammation and has been targeted with small‐molecule inhibitors (e.g., ST2825) in preclinical models [22]. *BMP2* has context‐dependent roles—it can suppress glioma stem‐like cell proliferation in some settings, consistent with its protective hazard ratio [23, 24]. *ADSL* is a key enzyme in purine biosynthesis and the fumarate node; although no direct ADSL inhibitor is available, the purine synthesis pathway is targeted by methotrexate and pemetrexed [25]. *ACADS*, *SOAT2*, and *ELANE* are less well studied in GBM; *ACADS* (short‐chain acyl‐CoA dehydrogenase) may affect mitochondrial fatty acid oxidation, and *SOAT2* is involved in cholesterol esterification—both could be explored with existing metabolic probes. Further experimental validation is required to assess their therapeutic potential.

Notably, our analysis revealed profound differences in the tumor immune microenvironment (TIME) between risk groups. The high‐risk group exhibited a more immunosuppressive and evasive phenotype, characterized by an increase in protumorigenic immune cells like neutrophils and resting NK cells, a decrease in activated NK cells, and a significant upregulation of a wide array of immune checkpoint molecules, chemokines, and immunomodulatory factors. This aligns with the enrichment of TGF‐*β* response and macrophage regulation in this group. These findings imply that the poor prognosis associated with the high‐risk score may be partly mediated by an immune‐cold and suppressive microenvironment, potentially explaining reduced responsiveness to immunotherapy. Consequently, the risk model could serve as a valuable tool for prescreening patients who might benefit from combination therapies targeting these specific immune escape mechanisms.

At the genomic level, the risk stratification was strongly associated with canonical GBM driver mutations. The near‐exclusive presence of *IDH1* mutations in the low‐risk group and the significant enrichment of *RB1* alterations in the high‐risk group are particularly striking. *IDH1*‐mutant gliomas are known to represent a distinct, more favorable prognostic subtype with a unique metabolic and epigenetic profile [26–29]. Their clustering within our low‐risk group validates the biological relevance of our signature. Conversely, *RB1* loss is a key event in cell cycle dysregulation and is associated with more aggressive disease in various cancers [30–33]. The mutual exclusivity pattern between these drivers in our cohort and the external MSK dataset reinforces the concept that our risk model captures fundamental biological divisions within GBM, separating *IDH1*‐driven, metabolically dysregulated tumors from *RB1*‐deficient, proliferative tumors. Notably, IDH1 mutations are rare in primary (de novo) GBM and define a distinct glioma subtype with a more favorable prognosis. In the TCGA‐GBM cohort, the IDH1‐mutant tumors (10.13% of low‐risk patients) likely represent either secondary GBM or, under the current WHO 2021 classification, astrocytoma, IDH‐mutant, CNS WHO Grade 4. Therefore, our risk model′s stratification may partly reflect this inherent molecular classification rather than independent prognostic value within primary IDH‐wildtype GBM.

The single‐cell RNA‐seq analysis provided crucial cellular and mechanistic insights. The specific enrichment of signature genes, particularly *ADSL* (a key enzyme in purine metabolism and the methyl cycle), within the malignant cell compartment underscores their tumor‐intrinsic role. The hdWGCNA coexpression network analysis in ADSL‐high malignant cells successfully deconvoluted a core transcriptional module (M4) correlated with this phenotype. The functional enrichment of this module in processes like neural precursor proliferation, gliogenesis, and RNA splicing directly links the metabolic state defined by our signature to transcriptional programs driving malignancy and stemness. Furthermore, the association with extracellular matrix and perineuronal net components hints at a role in remodeling the tumor niche to support invasion and progression.

Our study has several limitations. First, the sample size, especially for normal brain tissue controls, was relatively small, which may affect the robustness of differential expression calls for some genes. Second, although we employed computational deconvolution (CIBERSORT) and single‐cell data to infer immune and cellular contexts, these findings would be strengthened by orthogonal validation using multiplex immunohistochemistry (IHC) or flow cytometry on primary patient samples. Third, the mechanistic contributions of individual signature genes, particularly how they coordinately regulate propionate metabolism or interact with the identified upstream regulators (miRNAs and transcription factors), remain to be experimentally verified through functional assays in vitro and in vivo. Specifically, IHC on an independent GBM tissue microarray could validate the protein expression of key signature genes (e.g., ADSL and MYD88) and immune cell markers (CD8, CD68, and FOXP3) in high‐ versus low‐risk patients. Functional assays, including CRISPR‐based knockout or overexpression of *ADSL* or *MYD88* in GBM cell lines, followed by proliferation, migration, and immune coculture experiments, would help establish causality. Additionally, metabolomic profiling of propionate and its derivatives in patient samples could directly link the risk signature to propionate pathway activity.

In conclusion, we have established a novel seven‐gene signature derived from propionate metabolism that serves as an independent and reliable prognostic biomarker for GBM. This signature transcends a simple prognostic tool by illuminating a convergent biological theme: It bridges metabolic dysregulation, a suppressive immune microenvironment, specific genomic alterations (*IDH1* vs. *RB1*), and protumorigenic transcriptional networks at the single‐cell level. Our findings position propionate metabolism and its associated gene network as a pivotal axis in GBM aggressiveness. Future research should focus on experimentally dissecting the functional role of these genes and exploring whether targeting this metabolic‐immune axis could offer new therapeutic opportunities for high‐risk GBM patients.

## 5. Conclusions

Our study establishes a propionate metabolism‐based gene signature as an independent prognostic biomarker for GBM. Beyond prediction, our work elucidates that this signature encapsulates a high‐risk phenotype characterized by immunosuppression, distinct driver mutations (IDH1 vs. RB1), and malignant cell‐intrinsic transcriptional programs. This integrated perspective underscores the interplay between metabolism and the tumor ecosystem in driving GBM progression, offering a foundation for future mechanistic and therapeutic exploration.

## Author Contributions

Mengtong Li and Peng Lun: conception and design; Mengtong Li and Jiayi Liu: data analysis and interpretation; Xia Liu and Zichen Liu: writing—original draft; Mengtong Li and Peng Lun: writing—review and editing; Peng Lun: supervision.

## Funding

No funding was received for this manuscript.

## Ethics Statement

As this study is based on published or public datasets, it does not require ethical approval or consent.

## Consent

All authors consent to the publication of this study.

## Conflicts of Interest

The authors declare no conflicts of interest.

## Data Availability

The data that support the findings of this study are available from the corresponding authors upon reasonable request.
